# Pharmacokinetic Interaction of Green Rooibos Extract With Atorvastatin and Metformin in Rats

**DOI:** 10.3389/fphar.2019.01243

**Published:** 2019-10-23

**Authors:** Oelfah Patel, Christo J.F. Muller, Elizabeth Joubert, Bernd Rosenkranz, Malcolm J.C. Taylor, Johan Louw, Charles Awortwe

**Affiliations:** ^1^Biomedical Research and Innovation Platform (BRIP), South African Medical Research Council (SAMRC), Tygerberg, South Africa; ^2^Division of Clinical Pharmacology, Department of Medicine, Faculty of Medicine and Health Sciences, University of Stellenbosch, Tygerberg, South Africa; ^3^Division of Medical Physiology, Faculty of Health Sciences, Stellenbosch University, Tygerberg, South Africa; ^4^Department of Biochemistry and Microbiology, University of Zululand, KwaDlangezwa, South Africa; ^5^Plant Bioactives Group, Post-Harvest and Agro-Processing Technologies, Agricultural Research Council, Infruitec-Nietvoorbij, Stellenbosch, South Africa; ^6^Department of Food Science, Stellenbosch University, Matieland, South Africa; ^7^Central Analytical Facility, Mass Spectrometry Unit, Matieland, South Africa

**Keywords:** rooibos, atorvastatin, transporters, Wistar, rats

## Abstract

An aspalathin-rich green rooibos extract (Afriplex GRT™) has demonstrated anti-diabetic and hypolipidemic properties, while also moderately inhibiting CYP3A4 activity, suggesting a potential for herb–drug interaction. The present study, therefore, evaluated the effects of orally administered GRT on the pharmacokinetics of atorvastatin and metformin in Wistar rats. Wistar rats were orally treated with GRT (50 mg/kg BW), atorvastatin (40 mg/kg BW) or metformin (150 mg/kg BW) alone or 50 mg/kg BW GRT in combination with 40 mg/kg BW atorvastatin or 150 mg/kg BW metformin. Blood samples were collected at 0, 10, and 30 min and 1, 2, 4, 6, and 8 h and plasma samples obtained for Liquid chromatography-mass spectrometry (LC-MS/MS) analyses. Non-compartment and two-compartment pharmacokinetic parameters of atorvastatin and metformin in the presence or absence of GRT were determined by PKSolver version 2.0 software. Membrane transporter proteins, ATP-binding cassette sub-family C member 2 (*Abcc*2), solute carrier organic anion transporter family, member 1b2 (*Slco1b2*), ATP-binding cassette, sub-family B (MDR/TAP), member 1A (*Abcb1a*), and organic cation transporter 1 (*Oct1*) mRNA expression were determined using real-time PCR expression data normalized to β-actin and hypoxanthine-guanine phosphoribosyltransferase (HPRT), respectively. Co-administration of GRT with atorvastatin substantially increased the maximum plasma concentration (C_max_) and area of the plasma concentration–time curve (AUC_0-8_) of atorvastatin by 5.8-fold (*p* = 0.03) and 5.9-fold *(p = 0.02)*, respectively. GRT had no effect on the plasma levels of metformin. GRT increased Abcc2 expression and metformin downregulated Abcb1a expression while the combination of GRT with atorvastatin or metformin did not significantly alter the expression of Slco1b1 or Oct1 did not significantly alter the expression of *Sclo1b2* or *Oct1*. Co-administration of GRT with atorvastatin in rats may lead to higher plasma concentrations and, therefore, to an increase of the exposure to atorvastatin.

## Introduction

Diabetes mellitus (DM), characterized by failure to maintain physiological glucose and lipid levels, is a complex metabolic disorder that affects approximately 15.5 million people in Africa (International Diabetes Federation, 2017). Current DM treatment regimens frequently involve the use of multiple therapeutics, such as metformin combined with atorvastatin to control glycemia and dyslipidemia, thereby ensuring adequate metabolic control (Bell, 2004; Padwal et al., 2005; Matafome et al., 2011). Metformin, a biguanide anti-diabetic drug, enhances peripheral insulin sensitivity, resulting in lowered blood glucose levels through the reduction in hepatic glucose production (DeFronzo et al., 1991; Bailey and Turner, 1996; Krentz and Bailey, 2005). Atorvastatin is a lipid-lowering agent that competitively inhibits 3-hydroxy-3-methylglutaryl-CoA (HMG-CoA) reductase, thus reducing cholesterol levels and cardiovascular risk in type 2 diabetic patients (Pichandi et al., 2011; Guo and Nair, 2017; Norhammar et al., 2017).

Lately, therapeutics derived from natural products are becoming popular as alternative treatment strategies. These often include the use of extracts containing complex mixtures of bioactive phenolic compounds (Kang et al., 2012; Haddad et al., 2015; Oh, 2015; Shay et al., 2015). *Aspalathus linearis*, better known as rooibos, an indigenous South African fynbos legume shown to offer several health benefits, such as anti-inflammatory (Baba et al., 2009), anti-obesity (Sanderson et al., 2014), and more importantly, anti-diabetic (Muller et al., 2018) effects. The role of aspalathin as a major bioactive *C*-glucosyl dihydrochalcone in rooibos extracts was reviewed by Johnson et al. (2018). An aspalathin-enriched green rooibos extract (GRE) containing 18% aspalathin (Muller et al., 2012), as well as purified aspalathin has been reported to augment glucose uptake in L6 myotubes by activating 5′adenosine monophosphate-activated protein kinase (AMPK) and glucose transporter type 4 (GLUT 4) translocation to the cell membrane (Son et al., 2013; Kamakura et al., 2015). Aspalathin and GRE also demonstrated the ability to increase glucose uptake *via* GLUT 4 in palmitate-treated 3T3-L1 adipocytes and C2C12 muscle cells, thereby ameliorating palmitate-induced insulin resistance (Mazibuko et al., 2013; Mazibuko et al., 2015). In an *in vitro* study, a pharmaceutical-grade green rooibos extract (Afriplex GRT^™^), containing 12% aspalathin, has been shown to inhibit CYP3A4 activity (Patel et al., 2016), one of the CYPs responsible for metabolizing atorvastatin (Rowan et al., 2012). However, currently, there is a lack of *in vivo* evidence on the effect that rooibos extracts could have on the pharmacokinetics of prescribed chronic medications, specifically those used to treat diabetes and its related complications. This study, therefore, investigated the effect of GRT on the pharmacokinetics of atorvastatin and metformin in Wistar rats.

## Materials and Methods

### Plant Extract

The pharmaceutical-grade green rooibos extract, Afriplex GRT^™^ (GRT; 12% aspalathin), previously characterized in terms of major flavonoids and Z-2-(β-D-glucopyranosyloxy)-3-phenylpropenoic acid (PPAG) and investigated by Patel et al. (2016), was used in the current study.

### Materials and Reagents

Atorvastatin calcium (≥98%), sterile tissue culture water, dimethyl sulfoxide (DMSO), methanol, and HPLC grade acetonitrile and formic acid were purchased from Sigma-Aldrich (St. Louis, MO, United States). Metformin HCl 850 (> 99%; Sandoz SA Pty Ltd.) was purchased from MKEM Pharmacy (Bellville, South Africa).

### Instrumentation and Chromatographic Conditions

Liquid chromatographic separation of plasma samples (3 µL) was performed at 30ºC on a BEH C18 column (2.1 × 100 mm, 1.7 µm; Waters, Milford, United States), using a Waters Acquity Classic ultra-performance liquid chromatograph connected to a Waters Xevo TQ-MS mass spectrometer (Waters, Milford, MA, USA). Data were acquired using electrospray positive ionization in multiple reaction monitoring modes, with precursor-to-product ion transition of m/z 130 → 60.10 for metformin, m/z 559.50 → 440.40 for atorvastatin, and m/z 260.20 → 183.00 for propranolol (internal standard) with instrumental parameters as follows: capillary voltage 3.50 kV, desolvation temperature 400°C, and desolvation gas flow 800 L/h. The mobile phase consisted of 0.1% formic acid in water (A) and 0.1% formic acid in acetonitrile (B) delivered at a flow rate of 0.4 mL/min in gradient mode. The gradient started at 100% solvent A for 0.5 min and changed linearly to 100% B over 4 min. It then returned to initial conditions (100% A) after 4.1 min, with a total run time of 6 min to allow for column equilibration.

### Preparation of Standard Solutions and Quality Control Samples

Stock solutions of both atorvastatin and metformin standards were prepared by dissolving 50 mg of each compound in 50 ml of methanol. For each standard, 200 µl of the compound solution and 800 µl of acetonitrile were pipetted into an Eppendorf vial. The content of the vial was vortexed for 1 min and then centrifuged at 12,550 rpm for 3 min, whereafter an aliquot of the supernatant was transferred into an autosampler vial.

### Plasma Sample Preparation

Frozen plasma samples were thawed, allowed to equilibrate to room temperature and an aliquot (200 µl) vortexed after addition of 800 µl ice-cold acetonitrile, spiked with 1 µg/ml propranolol as an internal standard. Further processing was performed as described for the standards.

### Validation Procedures

#### Specificity

Plasma samples were spiked with 0.002 µg/ml atorvastatin and 0.002 µg/ml metformin and the chromatograms for atorvastatin and metformin in the absence and presence of blank rat plasma compared to evaluate specificity.

#### Linearity

The linearity interval tested was 0.002 to 0.1 µg/ml for atorvastatin and 0.002 to 0.1 µg/ml for metformin with replicate samples injected in triplicate. The linearity correlation coefficient (*r* ^2^) was estimated by regression analysis. The limit of detection (LOD) and limit of quantification (LOQ) were calculated as the lowest concentration of an analyte in a sample which yielded a signal-to-noise ratio of 3 (LOD) and 10 (LOQ), respectively. The baseline noise was estimated using MassLynx software (Waters, Milford, MA, United States).

#### Accuracy and Precision

Accuracy was determined for three concentrations (0.0075, 0.025, and 0.08 µg/ml) of atorvastatin or metformin with nine replicates at each concentration. Precision was expressed as repeatability (intra-day measurements) and intermediate precision (inter-day measurements). Plasma samples were spiked to give concentrations ranging from 0.002 to 0.1 µg/ml of atorvastatin or metformin with three replicates for each concentration.

### Pharmacokinetic Experiments in Rats

Twelve-week-old, male Wistar rats (weighing between 200 and 300 g) were housed at the Primate Unit and Delft Animal Centre (PUDAC) of the South African Medical Research Council (SAMRC) in a temperature-controlled room under a 24-h light/dark cycle, with food and water *ad libitum*. Approval for the study was granted by the Ethics Committee for Research on Animals (ECRA) of the South African Medical Research Council (ref. 04/15). All experiments were performed in accordance with the principles and guidelines of ECRA.

For pharmacokinetic studies, rats were randomly divided into six experimental groups (n = 24/group) and treated daily for 3 weeks through oral gavage. Treatment groups included: group 1, GRT (50 mg/kg BW); group 2, atorvastatin (40 mg/kg BW); group 3, metformin (150 mg/kg BW); group 4, combination of atorvastatin and GRT (atorvastatin, 40 mg/kg + GRT, 50 mg/kg BW); and group 5, combination of metformin and GRT (metformin, 150 mg/kg + GRT, 50 mg/kg BW). DMSO solution (0.01%) was used as vehicle control for untreated animals (Group 6). Body weight and blood glucose measurements *via* tail prick using a glucometer (One-Touch select; M-Kem Pharmacy, South Africa) were determined weekly. Rats were fasted overnight with free access to drinking water before dose administration. On day 21, blood samples (approximately 4 ml) were collected in heparinized tubes *via* the inferior vena cava at 0, 10, 30, min, and 1, 2, 4, 6, and 8 h post-treatment with GRT. Following blood collection, liver tissue was collected at 1, 2, 4, 6, and 8 h post-treatment with GRT. Rats were sacrificed by exsanguination using fluothane gas anesthesia. Plasma samples were obtained by centrifugation of the blood at 4000 rpm for 15 min and stored at −80°C for further analysis. Liver tissues were collected for molecular analysis.

### Quantitative Real-Time PCR Analyses

Total RNA was extracted from rat liver tissue collected at the 4- and 8-h post-treatment timepoints using the RNeasy kit (ThermoFischer Scientific Inc. Waltham, MA, United States). Briefly, tissues were homogenized using a TissueLyser (Qiagen GmbH, Hilden, Germany) at 13,500 rpm for 3 min, processed according to the manufacturer’s instructions and the extracted RNA was purified with RNeasy kit. RNA concentration and purity were quantified using a Nanodrop One spectrophotometer (Thermo Electron Scientific Instruments LLC, Madison, WI, United States). The RNA quality was determined using an Agilent 2100 Bioanalyzer (Agilent Technologies, Santa Clara, CA, United States). The Turbo DNase kit (ThermoFischer Scientific Inc) was used to remove genomic DNA as per the manufacturer’s recommendations. RNA samples were converted to cDNA using the High Capacity Reverse Transcription Kit (Applied Biosystems, Foster City, CA, United States) as recommended by the manufacturer. The mRNA levels of drug transporter genes; *Abcc2* (Rn00563231_m1), *Slco1b2* (Rn01492635_m1), *Abcb1a* (Rn01639253_m1), and *Oct 1* (Rn00562250_m1) were determined by performing quantitative real-time PCR using the ABI 7500 Instrument (ThermoFischer Scientific Inc.). Gene expression data were normalized to β-actin (Rn00667869_m1) and HPRT (Rn01527840_m1), respectively.

### Data Analyses

The plasma concentration versus time profiles of atorvastatin and metformin in the presence and absence of GRT were processed by PKSolver version 2.0 software (Pharsight, Mountain View, CA, USA). Non-compartment analysis was performed to estimate pharmacokinetic parameters, including the area under the plasma concentration–time curve (AUC_0-8_ and AUC_0-∞_) and apparent terminal half-life (T_1/2_), mean residence time (MRT), apparent (oral) volume of distribution (Vz/F) and clearance (CL/F), peak plasma concentration (C_max_), and the time to attain C_max_ (T_max_). Since the semi-logarithmic representation of the concentration–time data was well fitted by a straight line, it was appropriate to estimate terminal half-line as ln2/m, where m was the slope of regression line. In addition, apparent volume of the central or plasma compartment (V_1_), apparent volume of the peripheral compartment (V_2_), transfer rate constant from the central to peripheral compartment (K_12_), and transfer rate constant from the peripheral to central compartment (K_21_) were estimated using two-compartment model. Statistical comparisons between treatments were performed using GraphPad Prism^®^ version 7.03 (GraphPad Software Inc., San Diego, CA, United States). All data were expressed as mean ± SD. Comparisons between groups were performed using unpaired student t-test for PK parameters and one-way ANOVA with Dunnett’s multiple comparison test to compare mRNA gene expression with *p < 0.05* considered statistically significant.

## Results

### Effect of GRT, Atorvastatin, and Metformin, Alone or in Combination, on Body Weight and Blood Glucose Levels

Body weight and blood glucose levels were assessed weekly after daily oral administration of GRT, atorvastatin, and metformin, or combinations of GRT and the drugs for 3 weeks. After this treatment period, no significant changes (*p* ≥ 0.5) in body weight were observed. GRT monotherapy increased blood glucose levels, but the increase remained within the normal range (**Table 1**).

**Table 1:** Body weight and glucose levels of rats (n = 144) treated with GRT, atorvastatin alone, and combined with GRT and metformin alone and combined with GRT for 3 weeks.

Groups	Body weight	Glucose
**Control**	314.0 ± 25.4	4.2 ± 0.4
**Ator**	309.8 ± 30.3	4.3 ± 0.6
**Met**	311.0 ± 27.8	4.3 ± 0.6
**GRT**	322.2 ± 28.5	5.0 ± 0.8****
**GRT + Ator**	324.1 ± 39.7	4.2 ± 0.2
**GRT + Met**	316.9 ± 34.7	4.3 ± 0.3

Values are presented as the mean ± SD of body weight (g) and blood glucose (mmol/L), ****p < 0.0001. All treatment groups are compared to the control. Ator, atorvastatin; Met, metformin; GRT, green rooibos extract.

### LC-MS/MS Determination of Atorvastatin and Metformin

For the determination of atorvastatin and metformin in rat plasma, an LC-MS/MS method was developed and validated. Specificity was evaluated by comparing blank rat plasma to plasma spiked with atorvastatin or metformin. Representative chromatograms of atorvastatin, metformin, and propranolol (internal standard) are shown in **Supplementary Figures 1A–C**. Good recoveries were obtained for atorvastatin (97.9% ± 7.3%) and metformin (100.1% ± 0.5%) in the spiked samples at the 0.1 µg/ml level (**Table 2**). The lowest recoveries were 93.6 ± 6.6 for atorvastatin and 97.1 ± 3.5 for metformin, both at 0.005 µg/ml. The LOD values for metformin and atorvastatin were 0.001 and 0.002 µg/ml, respectively, while their LOQ values were 0.002 and 0.005 µg/ml, respectively. Accuracy values for the two analytes are presented in **Table 3**. The matrix effect was evaluated by comparing the peak areas of the analytes spiked into post-treated blank plasma samples with those for analytes spiked into the mobile phase at the same concentrations. No matrix effects were detected as no significant suppression or enhancement of peak area under the current chromatographic, and mass spectrometry conditions were observed.

**Table 2:** Mean recovery, repeatability, and intermediate precision for atorvastatin and metformin in rat plasma.

Compounds	Nominal concentration (µg/ml)	Determined concentration (µg/ml)	Repeatability	% Recovery	Intermediate Precision
Atorvastatin	0.002	0.002 ± 0.1	8.5 ± 1.8	100.0 ± 9.4	10.6
	0.005	0.005 ± 0.3	4.8 ± 0.9	93.6 ± 6.6	7.2
	0.01	0.010 ± 0.3	2.2 ± 1.2	103.0 ± 3.4	7.7
	0.02	0.021 ± 0.6	5.0 ± 2.9	102.9 ± 6.5	2
	0.05	0.052 ± 0.3	1.1 ± 0.4	103.1 ± 1.4	3.8
	0.1	0.098 ± 2.5	6.4 ± 2.7	97.9 ± 7.3	5.4
Metformin	0.002	0.002 ± 0.2	8.6 ± 3.4	104.2 ± 14.3	0.7
	0.005	0.005 ± 0.2	1.8 ± 0.7	97.1 ± 3.5	3.6
	0.01	0.010 ± 0.1	1.3 ± 0.8	98.9 ± 1.7	1.9
	0.02	0.020 ± 0.2	1.5 ± 0.4	99.3 ± 1.9	1.5
	0.05	0.050 ± 0.5	0.5 ± 0.3	100.4 ± 1.0	1.4
	0.1	0.100 ± 0.4	0.3 ± 0.1	100.1 ± 0.5	8.6

**Table 3:** Accuracy data for QC samples (spiked rat plasma).

	Metformin (n = 9)		Atorvastatin (n = 9)	
	Measured Conc	Accuracy	Measured Conc	Accuracy
**Low spike 7.5 μg/L**	7.3	97.7	6.9	91.5
**Med spike 25 μg/L**	25.3	101.2	25.4	101.5
**High spike 80 μg/L**	80.3	100.3	83.2	104.1

### Pharmacokinetic Analysis of Atorvastatin and Metformin

**Figure 1:**
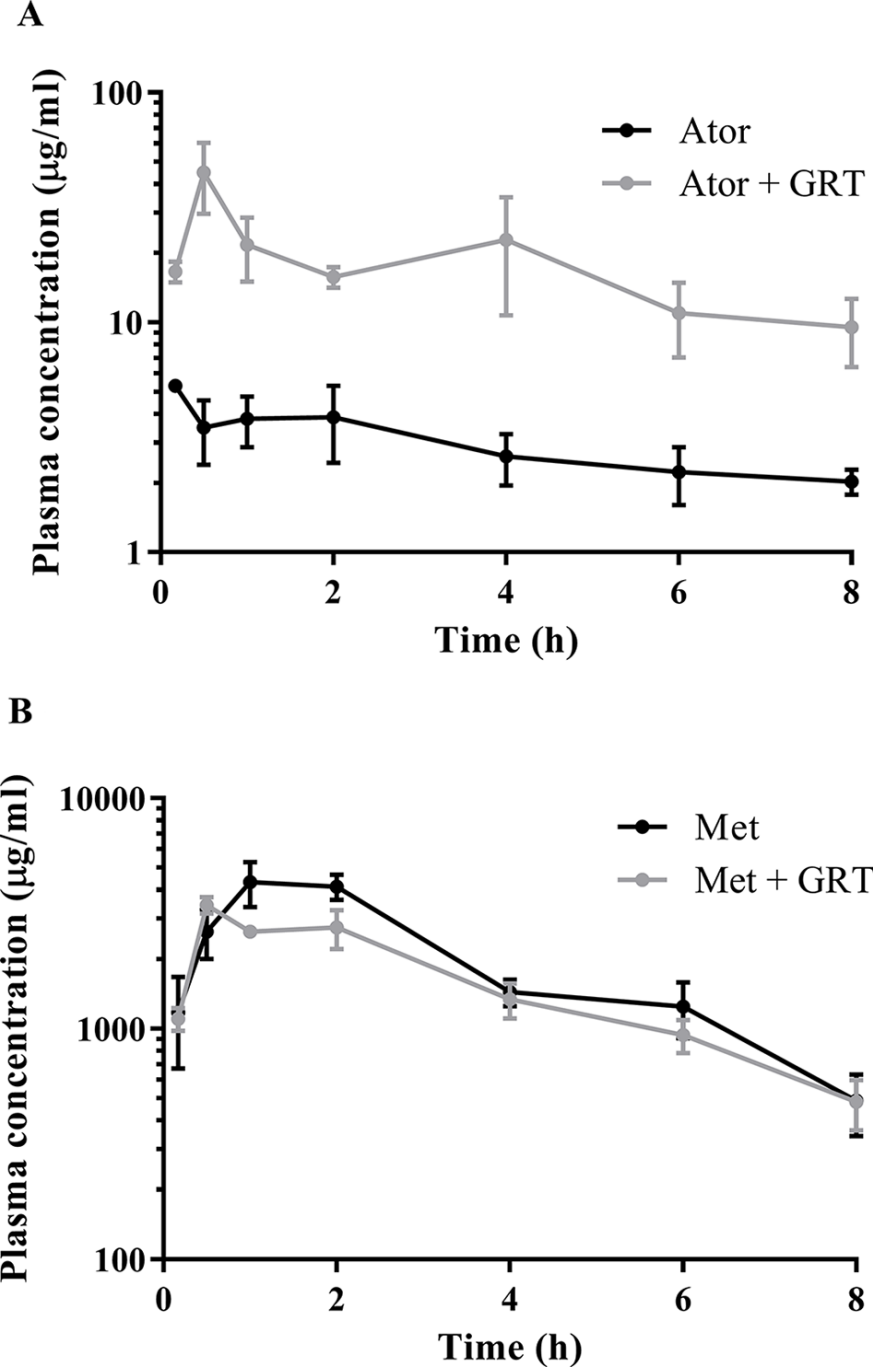
Semi-log plot of the plasma concentration versus time of **(A)** atorvastatin and **(B)** metformin in the absence and presence of GRT in Wistar rats (n = 24 per group). The black line indicates atorvastatin or metformin, and gray lines indicate atorvastatin with GRT and metformin with GRT. Ator, atorvastatin; Met, metformin; GRT, green rooibos extract.

The plasma concentration versus time profiles of the drugs alone and in combination with GRT, after oral administration, are depicted in **Figures 1A, B**. Pharmacokinetic parameters of atorvastatin and metformin alone, as well as in combination with GRT, are summarized in **Table 4**. Concomitant intake of GRT increased the plasma concentration of atorvastatin (**Figure 1A**). C_max_ of atorvastatin in combination with GRT was increased by 5.8-fold when compared to atorvastatin given alone (from 8.42 ± 3.88 µg/ml to 48.72 ± 20.38 µg/ml; *p* = 0.03). Similarly, the AUC_0-8_ was significantly increased by 5.9-fold (from 23.16 ± 0.62 μg·ml^−1^·h^−1^ to 135.66 ± 53.24 μg·ml^−1^·h^−1^; *p* = 0.02; **Table 4**). Both CL/F, and Vz/F values of atorvastatin were significantly decreased by 78% and 83% in the presence of GRT. A significant reduction in the apparent volume of the peripheral compartment, V_2_, and a decrease of k_12_ were observed for atorvastatin when co-administered with GRT (*p* < 0.01). The plasma concentrations of metformin did not change significantly when co-administered with GRT (**Figure 1B**), and none of the pharmacokinetic parameters was significantly affected (**Table 4**).

**Table 4:** Pharmacokinetic parameters of atorvastatin (40 mg/kg) and metformin (150 mg/kg) alone or in combination with GRT (50 mg/kg) in Wistar rats (n = 144).

Pharmacokinetic parameters	Ator	Ator + GRT	Met	Met + GRT
*T*_max_ (h)	0.78 ± 1.06	0.67 ± 0.29	1.67 ± 0.58	1.00 ± 0.87
*C*_max_ (µg/mL)	8.42 ± 3.88	48.72 ± 20.38*	4, 727.97 ± 1471.97	3, 530.64 ± 517.61
*T*_1/2_ (h)	4.60 ± 1.97	3.12 ± 1.31	2.38 ± 0.82	2.75 ± 1.15
AUC_0-8_ (µg/mL*h)	23.16 ± 0.62	135.66 ± 53.27*	15, 983.48 ± 3529.52	12, 538.03 ± 1919.92
AUC_0-∞_ (µg/mL^*^h)	37.47 ± 9.48	176.27 ± 67.75	17, 856.48 ± 4797.19	14, 667.16 ± 2696.19
MRT_0-∞_ (h)	7.71 ± 2.81	5.48 ± 1.64	3.78 ± 0.67	4.29 ± 1.36
Vz/F (mg/kg)/(L/kg)	6.88 ± 1.28	1.14 ± 0.55**	0.03 ± 0.01	0.04 ± 0.01
CL/F (mg/kg)/(L/kg*hr)	1.11 ± 0.27	0.25 ± 0.06**	0.01 ± 0.00	0.01 ± 0.00
V_1_ (mg/kg)/(μg/mL)	2.96 ± 2.67	0.37 ± 0.40	0.02 ± 0.01	0.03 ± 0.01
V_2_ (mg/kg)/(μg/mL)	22.61 ± 21.00	1.66 ± 0.93**	0.06 ± 0.10	0.01 ± 0.02
K_12_ (1/h)	33.70 ± 57.23	7.06 ± 9.94	0.41 ± 0.45	0.45 ± 0.78
K_21_ (1/h)	0.40 ± 0.38	0.38 ± 0.38	0.37 ± 0.29	0.69 ± 0.49

Data are the mean ± SD (n = 24 per group). ^*^ p < 0.05; ^**^ p < 0.01, compared to atorvastatin with GRT and atorvastatin alone.

T_max_, time to reach maximum (peak) plasma concentration; C_max_, maximum (peak) plasma drug concentration; T_1/2_, elimination half-life; AUC_0-8_, area under the plasma concentration–time curve from time zero to time 8; AUC_0-∞_, area under the plasma concentration–time curve from time zero to infinity; MRT_0-∞_, mean residence time from time zero to infinity; Vz/F, apparent volume of distribution during terminal phase after non-intravenous administration; CL/F, apparent total clearance of the drug from plasma after oral administration; V_1_, apparent volume of the central or plasma compartment in a two-compartment model; V_2_, apparent volume of the peripheral compartment in a two-compartment model; K_12_, transfer rate constant from the central to peripheral compartment; K_21_, transfer rate constant from the peripheral to central compartment.

### mRNA Expression of Drug Transporters

To gain an insight into the molecular mechanism of the potential interaction of GRT with metformin and/or atorvastatin *via* drug transporters in the liver samples of treated rats, we measured the mRNA levels of *Abcc2*, *Slco1b2*, *Abcb1a*, and *Oct1* genes. Treatment with GRT alone increased the expression of *Abcc2* (*p* < 0.05, **Table 5**). Metformin downregulated the expression of *Abcb1a* (*p* < 0.0001, **Table 5**) which was sustained by the addition of GRT. No significant effects on the expression of *Slco1b2* or *Oct1* were observed across treatment groups (**Table 5**).

**Table 5:** Relative gene expression of GRT alone and combined with atorvastatin and metformin.

	*Abcc2*		*Slco1b2*		*Abcb1a*		*Oct1*	
	4 h	8 h	4 h	8 h	4 h	8 h	4 h	8 h
Control	1.04 ± 0.00	0.96 ± 0.25	1.11 ± 0.19	0.96 ± 0.24	1.07 ± 0.27	1.13 ± 0.07	0.70 ± 0.13	1.03 ± 0.18^#^
Ator	0.99 ± 0.27	1.01 ± 0.29	0.81 ± 0.21	1.01 ± 0.14	0.97 ± 0.06	0.89 ± 0.25	0.49 ± 0.05	0.82 ± 0.05
Met	0.49 ± 0.01	0.78 ± 0.11	0.79 ± 0.30	0.50 ± 0.21	0.66 ± 0.07	0.34 ± 0.10***	0.75 ± 0.18	0.82 ± 0.21
GRT	1.70 ± 0.49	1.63 ± 0.06*	1.14 ± 0.28	1.13 ± 0.00	1.03 ± 0.20	1.15 ± 0.07	0.67 ± 0.12	1.18 ± 0.19
Ator + GRT	1.28 ± 0.16	1.12 ± 0.20	1.03 ± 0.17	2.22 ± 2.06	1.39 ± 0.65	2.27 ± 1.86	0.39 ± 0.86	1.03 ± 0.26
Met + GRT	1.88 ± 1.79	1.45 ± 0.38	1.24 ± 0.27	0.78 ± 0.35	1.01 ± 0.51	0.60 ± 0.28**	2.40 ± 2.23	1.16 ± 0.17

Gene expression levels are presented relative to *β*-actin and HPRT. Liver tissue was collected at 4- and 8-h timepoints, respectively. Data are the mean ± SD (n = 3 animals per group/timepoint). ^*^p < 0.05; ^**^p < 0.01; ^***^p < 0.0001 compared to the control. ^#^p < 0.05 compared to the 4-h timepoint.

## Discussion

Clinical case studies have highlighted possible herb–drug interactions of rooibos with hypolipidemic medications, such as statins resulting in elevated liver enzymes and toxicity (Sinisalo et al., 2010; Reddy et al., 2016). The current study was performed to assess the effect of GRT on the *in vivo* pharmacokinetics of atorvastatin and metformin in Wistar rats.

GRT contains high levels of dihydrochalcones (aspalathin and nothofagin, 7400 µg/50 mg dose), flavonols (quercetin-3-*O*-robinobioside, isoquercitrin, rutin, and hyperoside, 1250 µg/50 mg dose) and flavones (orientin, isoorientin, vitexin, and isovitexin, 1650 µg/50 mg dose) (Patel et al., 2016). These constituents may possibly affect the bioavailability of atorvastatin. Quercetin, the aglycone of the quantified rooibos flavonols, has modulatory effects on function and expression of CYP3A4 in rats, and inhibits P-glycoprotein (P-gp) ATPase, as well as P-gp–mediated efflux of ritonavir in Caco-2 cells (Cho and Yoon, 2015). *In vivo*, quercetin has been shown to increase the plasma concentrations of CYP3A4 substrates, such as pioglitazone (Ming et al., 2008; Umathe et al., 2008), rosiglitazone (Kim et al., 2005), and rivastigmine (Palle and Neerati, 2017).

The significant increase in the plasma concentration of atorvastatin, when co-administered with GRT, could be due to an effect on absorption and/or metabolism of atorvastatin. Co-administration of GRT was associated by a similar percentage decrease of Vz/F and CL/F (78% and 83%), confirming an increase in the bioavailability (F) of atorvastatin in the presence of GRT. This could be due to a possible interaction *via* drug metabolizing and transporter genes in the enterocytes and/or in the hepatocytes.

We have previously shown that GRT moderately inhibits CYP3A4 activity *in vitro* (Patel et al., 2016), which can affect the bioavailability of drugs metabolized by this CYP iso-enzyme, such as atorvastatin (Palle and Neerati, 2017). Since the bioavailability of atorvastatin is ca. 14% (Lennernäs, 2003; Khan and Dehghan, 2011), a significant increase through inhibition of CYP3A4 by GRT could explain the increased plasma concentrations. Elevated plasma drug concentrations, C_max_ and AUC *in vivo* correlates with the *in vitro* effect of GRT on the inhibition of CYP3A4, resulting in a decrease of the high intestinal clearance and first-pass metabolism of atorvastatin (Lennernäs, 2003). The compartmental pharmacokinetic analysis confirmed the decrease of the apparent peripheral volume of distribution. The lack of effect of GRT on K_21_ is due to the fact that the transfer from the peripheral to the central compartment is not expected to be affected by pharmacokinetic interaction.

Concomitant administration of GRT with metformin did not have an effect on metformin plasma concentrations in the present study. Previous studies showed that polyphenols widely distributed in medicinal plants can affect the oral bioavailability of metformin (Kamble et al., 2016). Pomegranate juice significantly reduced the efficacy of metformin in rats (Awad et al., 2016). In contrast to these findings, the concomitant administration of GRT with metformin *in vivo* did not affect the plasma concentration of metformin, suggesting that interaction of GRT (rooibos) with metformin is unlikely. Although metformin is not dependent on cytochrome P450 (CYP450) metabolism, drug transporting membrane proteins, such as the solute carrier family of transporters, including organic cation transporters (OCTs), and ATP-binding cassette transporters alters the extent of its pharmacological effects (Mato et al., 2018; Wu et al., 2018). GRT alone significantly increased *Abcc2* mRNA expression, while in combination with metformin or atorvastatin, no significant effect was demonstrated. Metformin monotherapy reduced *Abcb1a* mRNA expression, and in combination with GRT, *Abcb1a* mRNA expression remained downregulated. These findings suggest that GRT does not substantially interfere with the major drug transporters involved in the pharmacokinetics of metformin and atorvastatin. The role of hepatocellular uptake and efflux cell membrane transporters is important in the metabolic clearance of drugs (Shitara et al., 2006; Shugarts and Benet, 2009; Lusin et al., 2016; Kimoto et al., 2018). Specifically, the metabolism and elimination of statins are largely dependent on the ability of the liver to uptake, metabolize, and clear these drugs (Fong, 2016). Statins are both substrates and inhibitors of *Abcc2*, suggesting that statin-mediated hepatotoxic effects may result from the inhibition of these efflux transporters. However, the genes measured in this study does not explain the observed changes in atorvastatin pharmacokinetics. Atorvastatin is a substrate for organic anion transporting polypeptide 1b1 (oatp1b1 [human]) and in rats mediated primarily through *Slco1b2* prior to undergoing extensive metabolism by CYP3A (Chang et al., 2014). In a previous study, we have shown that GRT moderately inhibits CYP3A4 activity using a Vivid^®^ recombinant CYP3A4 enzymatic assay (Patel et al., 2016).

This *in vivo* non-clinical study showed a significant pharmacokinetic interaction between GRT and atorvastatin, suggesting that the interaction of GRT may affect the safety and efficacy profile of this drug. Although further studies are required to elucidate the precise mechanism of pharmacokinetic interference, patients on atorvastatin should be cautioned for possible herb–drug interaction when consuming rooibos products.

## Data Availability Statement

All datasets generated for this study are included in the manuscript/**Supplementary Files**.

## Ethics Statement

This study was carried out in accordance with the recommendations of the Ethics Committee for Research on Animals (ECRA) of the South African Medical Research Council (ref. 04/15). The protocol was approved by the Ethics Committee for Research on Animals (ECRA) of the South African Medical Research Council.

## Author Contribution

OP, CM, EJ, BR, and CA participated in research design. OP, MT, and CA conducted laboratory experiments. OP, CM, and CA contributed new reagents or analytical tools. OP, CM, MT, and CA performed data analysis. OP, CM, EJ, BR, MT, JL, and CA wrote or contributed to the writing of the manuscript.

## Funding

This work was supported in part by the National Research Foundation (NRF) Thuthuka Programme (Grant 99381) and the Biomedical Research and Innovation Platform of the South African Medical Research Council. Afriplex GRT^™^ was provided by Afriplex, Paarl, South Africa.

## Conflict of Interest

The authors declare no conflict of interest. The founding sponsors had no role in the design of the study; in the collection, analyses, or interpretation of data; in the writing of the manuscript, and in the decision to publish the results.

The authors declare that the research was conducted in the absence of any commercial or financial relationships that could be construed as a potential conflict of interest.

## Abbreviations

AUC, area under the curve; AMPK, AMP-activated protein kinase; BW, body weight, Cl/F, systemic plasma clearance following oral administration of a xenobiotic; C_max_, peak plasma concentration; CYPs, cytochrome P450 enzymes; DM, diabetes mellitus; DMSO, dimethyl sulfoxide; F, bioavailability; GRE, green rooibos extract; GRT, Afriplex green rooibos extract; HMG-CoA, 3-hydroxy-3-methylglutaryl-CoA; LC-MS/MS, liquid chromatography-tandem mass spectrometry; LOD, limit of detection; LOQ, limit of quantification; MRT, mean residence time; P-gp, P-glycoprotein; PPAG, *Z*-2-(β-D-glucopyranosyloxy)-3-phenylpropenoic acid; QC, quality control; t_1/2_, half-life; *T*_max_, time to attain C_max_; Vz/F, apparent (oral) volume of distribution; β-actin, beta-actin; HPRT, hypoxanthine-guanine phosphoribosyltransferase; *Oct1*, organic cation transporter 1; *Slo1b2*, solute carrier organic anion transporter family, member 1b2; *Abcb1a*, ATP-binding cassette, sub-family B (MDR/TAP), member 1A; *Abcc2*, ATP-binding cassette subfamily C member 2.
