# Supplementary figures and images for: Pharmacokinetic Interaction of Green Rooibos Extract With Atorvastatin and Metformin in Rats
Source: Front Pharmacol. 2019 Oct 23;10:1243. doi: 10.3389/fphar.2019.01243 (PMC6822546; doi:10.3389/fphar.2019.01243)

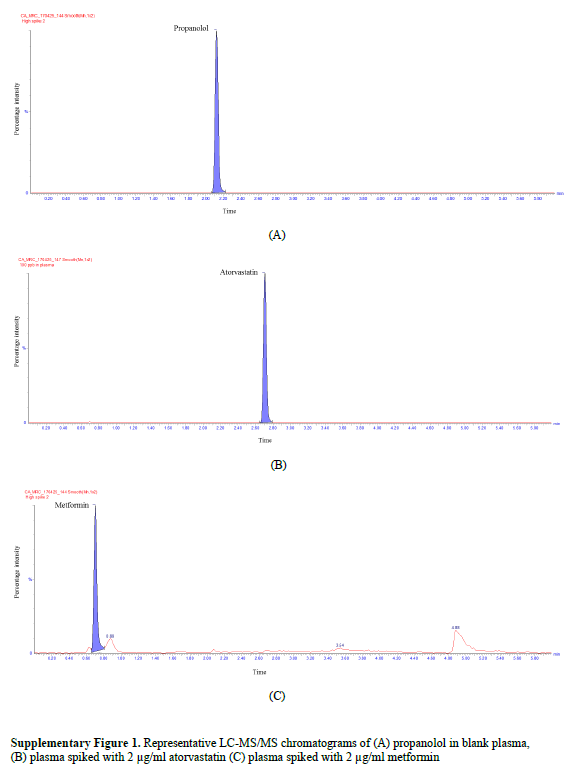

Supplement: Supplementary file 1 [file Image_1.tif]
